# Understanding the Impact of Brain Disorders: Towards a ‘Horizontal Epidemiology’ of Psychosocial Difficulties and Their Determinants

**DOI:** 10.1371/journal.pone.0136271

**Published:** 2015-09-09

**Authors:** Alarcos Cieza, Marta Anczewska, Jose Luis Ayuso-Mateos, Mary Baker, Jerome Bickenbach, Somnath Chatterji, Sally Hartley, Matilde Leonardi, Tuuli Pitkänen, PARADISE Consortium

**Affiliations:** 1 Faculty of Social and Human Sciences, School of Psychology, University of Southampton, Southampton, United Kingdom; 2 Department of Medical Informatics, Biometry and Epidemiology (IBE), Chair for Public Health and Health Services Research, Research Unit for Biopsychosocial Health, Ludwig-Maximilians-University (LMU), Munich, Germany; 3 Swiss Paraplegic Research, Nottwil, Switzerland; 4 Department of Psychiatry, Institute of Psychiatry and Neurology, Warsaw, Poland; 5 Instituto de Salud Carlos III, Centro de Investigación Biomédica en Red de Salud Mental (CIBERSAM), Department of Psychiatry, Universidad Autónoma de Madrid, Psychiatry Service, Instituto de Investigación del Hospital Universitario de La Princesa (IIS-IP), Madrid, Spain; 6 European Brain Council, Brussels, Belgium; 7 Multi-Country Studies, Department of Measurement and Health Information Systems, World Health Organization, Geneva, Switzerland; 8 Faculty of Medicine and Health Sciences, University of East Anglia, Norwich, United Kingdom; 9 Faculty of Health Sciences, University of Sydney, Sydney, New South Wales, Australia; 10 The London School of Hygiene and Tropical Medicine, London, United Kingdom; 11 Neurology, Public Health and Disability Unit, Scientific Directorate, Neurological Institute Carlo Besta IRCCS Foundation, Milan, Italy; 12 A-Clinic Foundation, Helsinki, Finland; The University of Queensland, AUSTRALIA

## Abstract

**Objective:**

To test the hypothesis of ‘horizontal epidemiology’, i.e. that psychosocial difficulties (PSDs), such as sleep disturbances, emotional instability and difficulties in personal interactions, and their environmental determinants are experienced in common across neurological and psychiatric disorders, together called brain disorders.

**Study Design:**

A multi-method study involving systematic literature reviews, content analysis of patient-reported outcomes and outcome instruments, clinical input and a qualitative study was carried out to generate a pool of PSD and environmental determinants relevant for nine different brain disorders, namely epilepsy, migraine, multiple sclerosis, Parkinson’s disease, stroke, dementia, depression, schizophrenia and substance dependency. Information from these sources was harmonized and compiled, and after feedback from external experts, a data collection protocol including PSD and determinants common across these nine disorders was developed. This protocol was implemented as an interview in a cross-sectional study including a convenience sample of persons with one of the nine brain disorders. PSDs endorsed by at least 25% of patients with a brain disorder were considered associated with the disorder. PSD were considered common across disorders if associated to 5 out of the 9 brain disorders and if among the 5 both neurological and psychiatric conditions were represented.

**Setting:**

The data collection protocol with 64 PSDs and 20 determinants was used to collect data from a convenience sample of 722 persons in four specialized health care facilities in Europe.

**Results:**

57 of the PSDs and 16 of the determinants included in the protocol were found to be experienced across brain disorders.

**Conclusion:**

This is the first evidence that supports the hypothesis of horizontal epidemiology in brain disorders. This result challenges the brain disorder-specific or vertical approach in which clinical and epidemiological research about psychosocial difficulties experienced in daily life is commonly carried in neurology and psychiatry and the way in which the corresponding health care delivery is practiced in many countries of the world.

## Introduction

Psychosocial difficulties (PSDs), such as sleep disturbances, emotional instability and difficulties in personal interactions and in work are prevalent in people with neurological and psychiatric disorders–which we here call ‘brain disorders’. PSDs are commonly used to estimate disease severity in neurological disorders [1]. In psychiatric disorders they are also used as diagnostic criteria [2]. Information about the PSDs people with brain disorders experience is as a result routinely obtained both in clinical practice and research.

Information about PSDs—whether it comes from clinical practice or research—is typically reported for each brain disorder in particular and only in exceptional cases are comparisons across brain disorders made. In a multi-method review, Wittchen et al. tellingly found that only with very few exceptions European studies concentrate on single disorders [3]. They found no study that systematically examined the difficulties associated with the full range of brain disorders. Where comparisons across brain disorders are targeted, these are usually restricted to single PSD, mostly body functions such as fatigue [4] or pain [5].

A focus on single conditions, in a silo-like manner is in some cases justifiable and necessary. For example, biomedical research must focus on the differential brain mechanisms that cause condition-specific symptoms. Similarly, life course approaches targeting shedding light on the epidemiology of different disorders, i.e. risk factors for depression, or on disorder-specific outcomes, i.e. impact of anxiety disorders in the adolescence on education achievement, are inevitably disorder-specific [6, 7]. The question is whether a disease-specific approach is also appropriate when identifying, understanding the impact of and treating the PSDs that people with brain disorders experience in their daily lives.

There is no good reason to believe that PSDs are fundamentally different between brain disorders, especially if we operationalize PSDs in terms of the World Health Organisation’s *International Classification of Functioning*, *Disability and Health* (ICF) [8]. Based on its conceptual model of functioning and disability, impairments in body functions and structures, activity limitations and participation restrictions are not a direct consequence of biological mechanisms of the health condition, but rather an outcome of the interaction between those mechanisms and features of the person’s environment and personal factors [8].

Relying on this understanding, we hypothesize that there are PSDs—defined as impairments of mental functions and impairments of body functions under central nervous system control, activity limitations and participation restrictions—that are common in people with brain disorders despite variations in symptomatology, aetiology, and the biochemical basis of their disorders.

There are several reasons that intuitively support the hypothesis of commonalities in PSDs across brain disorders. People with different brain disorders are subject to similar physical, social, and attitudinal influences, such as the built environment, support of the family, attitudes about their illnesses, and views about personal responsibility. Even though intensity can vary, the kinds of PSDs people experience in their lives, and which are influenced by these factors, are probably similar. Clinicians working with patients with neurological and psychiatric disorders are familiar with the fact that there are some common difficulties that their patients experience, such as sleep problems, difficulties with affect, maintaining personal relationships and holding a job. There is also research that supports this clinical experience [4, 5, 9–11]. Finally, it is well known that co-morbidity within brain disorders is very high, making it more intuitive to detach PSD from specific brain disorders [3].

If common PSDs are experienced by people with different brain disorders, it is reasonable to assume that there are also cross-cutting determinants of these PSDs. Using these determinants as a basis for developing interventions or strategies targeting PSDs across brain disorders would be more cost-effective than is currently the case. In fact, promising cross-cutting strategies are already recommended for instance for improving the experience of care for people using mental health services [12]. Lessons could be learned from the effectiveness of such strategies. The marginal utility of investing in common interventions and strategies further increases their cost-effectiveness to society.

The project called PARADISE (Psychosocial fActors Relevant to BrAin DISorders in Europe, www.paradiseproject.eu) funded under the FP7 by the European Commission, gave us the opportunity to test the hypothesis of commonalities across brain disorders. The PARADISE consortium was composed of 10 partners across Europe with clinical, epidemiological and public health expertise. Patients’ organizations were informed about the project and involved in it through the European Brain Council. In the present study we use a multi-method approach to test the hypothesis that people with different brain disorders experience PSDs and determinants in common. This hypothesis was called ‘horizontal epidemiology’ to highlight the difference from the standard, brain disorder-specific approach.

## Material and Methods

### Ethics statement

All study-relevant documentation and the written informed consent forms provided to the participants were approved by the Ethics Committees of the Neurological Institute Carlo Besta IRCCS Foundation in Milan, Italy, the Institute of Psychiatry and Neurology in Warsaw, Poland, the teaching hospital La Princesa of the University of Madrid in Madrid, Spain and the Järvenpää Addiction Hospital in Haarajoki, Finland. The present study has therefore been performed in accordance with the ethical standards laid down in the 1964 Declaration of Helsinki and its later amendments.

The present study has two parts. In the first one, PSDs and their determinants relevant across brain disorders were identified, while in the second part the hypothesis of ‘horizontal epidemiology’ was challenged. Each part is described in detail as follows.

### Identifying PSDs and their determinants relevant across brain disorders

To challenge the hypothesis of horizontal epidemiology we first needed to select brain disorders sufficiently diverse in terms of aetiology, biochemical bases, signs and symptoms. The selected brain disorders were: dementia, depression, epilepsy, migraine, multiple sclerosis (MS), Parkinson´s disease (PD), schizophrenia, stroke and substance dependency. These disorders have been selected for: 1) Being among the most burdensome disorders according to the Global Burden of Disease study [13]; 2) Occurring throughout Europe and across demographic and socio-economic categories; 3) Including disorders that have both intermittent (epilepsy and depression) and continuous (dementia and substance use disorders) symptomatology and consequences.

**Fig 1:**
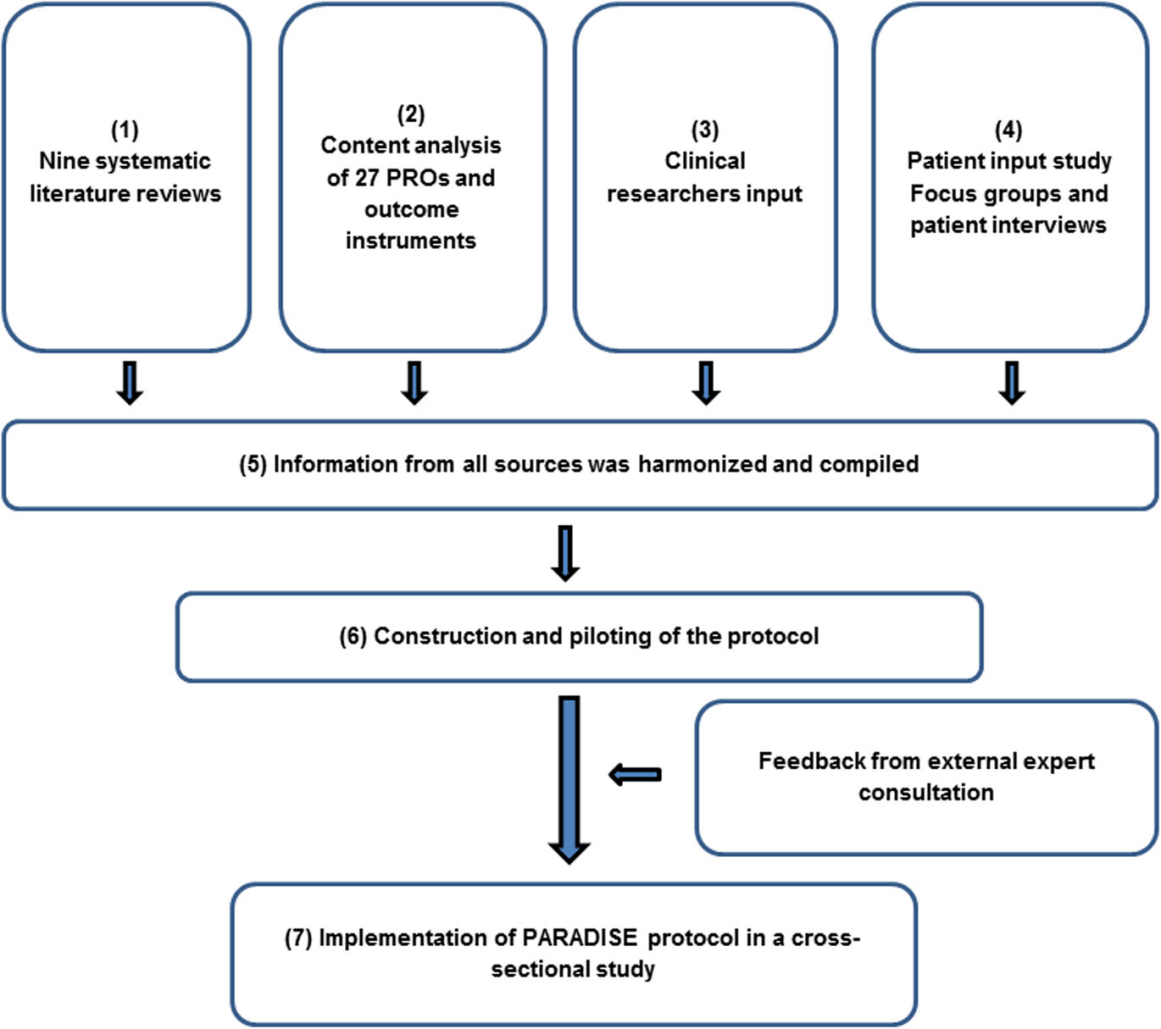
PARADISE approach with literature reviews, content analysis of outcome instruments, clinical input and qualitative study. PARADISE multi-method approach with systematic literature reviews, content analysis of patient-reported outcomes (PROs) and outcome instruments, clinical input and a qualitative study. Information from all sources was harmonized and compiled and a data collection protocol developed also including feedback from an external expert consultation. The protocol included all potentially relevant PSDs and their determinants across brain disorders. This protocol was implemented in a cross-sectional study.

Secondly, we followed a multi-method approach depicted in Fig 1: (1) systematic literature reviews, (2) content analysis of patient-reported outcomes (PROs) and outcome instruments, (3) clinical input and (4) a qualitative study. Information from all sources was (5) harmonized and compiled and (6) a data collection protocol developed also including feedback from an external expert consultation. The protocol included all potentially relevant PSDs and their determinants across brain disorders. (7) This protocol was implemented in a cross-sectional study.

### (1) Systematic literature reviews

Nine systematic literature reviews, one for each brain disorder were carried out, and have been published separately [14–18]. The literature between 2005 and 2010 was searched to identify PSDs and their determinants reported in intervention studies, longitudinal observational and qualitative studies (S1 File, S2 File). The PSDs and determinants reported were extracted as well as all Patient Reported Outcomes (PROs) and any other outcome instrument used. A detail description of the approach followed in the PARADISE literature reviews is presented in Świtaj et al. [15].

### (2) PROs

Clinical researchers who carried out the brain disorder-specific reviews selected the three PROs or outcome instruments that, in their experience treating patients with the disorders in the project, best captured the PSDs their patients experienced. Twenty-seven PROs and outcome instruments were selected.

### (3) Clinical input

The clinical researchers, on the basis of the results of their literature reviews, and their own clinical expertise, provided for each brain disorder determined which PSDs were the most salient for their patients.

### (4) Qualitative study

Focus groups and patient interviews gathered information about PSDs and their determinants. One focus group was conducted for each brain disorder, with three exceptions: for substance dependency two gender-specific groups were held to facilitate the discussion of gender-specific issues; two focus groups were conducted for stroke to accommodate different cognitive levels of stroke patients; and for dementia individual interviews were conducted, or, for very severely compromised patients, interviews with their caregivers were substituted.

### (5) Compilation and selection for data collection protocol

The extracted PSDs and determinants from the literature reviews and the qualitative study were grouped by underlying concept and named using ICF terms during a two-day workshop, in which all researchers participated. The items or content of the 27 PROs and outcome measures as well as the PSDs and determinants resulting from the clinical input were also grouped using ICF terms after applying standardized linking rules described by Cieza et al. [19]. Using the ICF as a standard language made it possible to compare PSDs and determinants from the literature reviews, PROs and outcome measures, the expert input and the qualitative studies.

**Fig 2:**
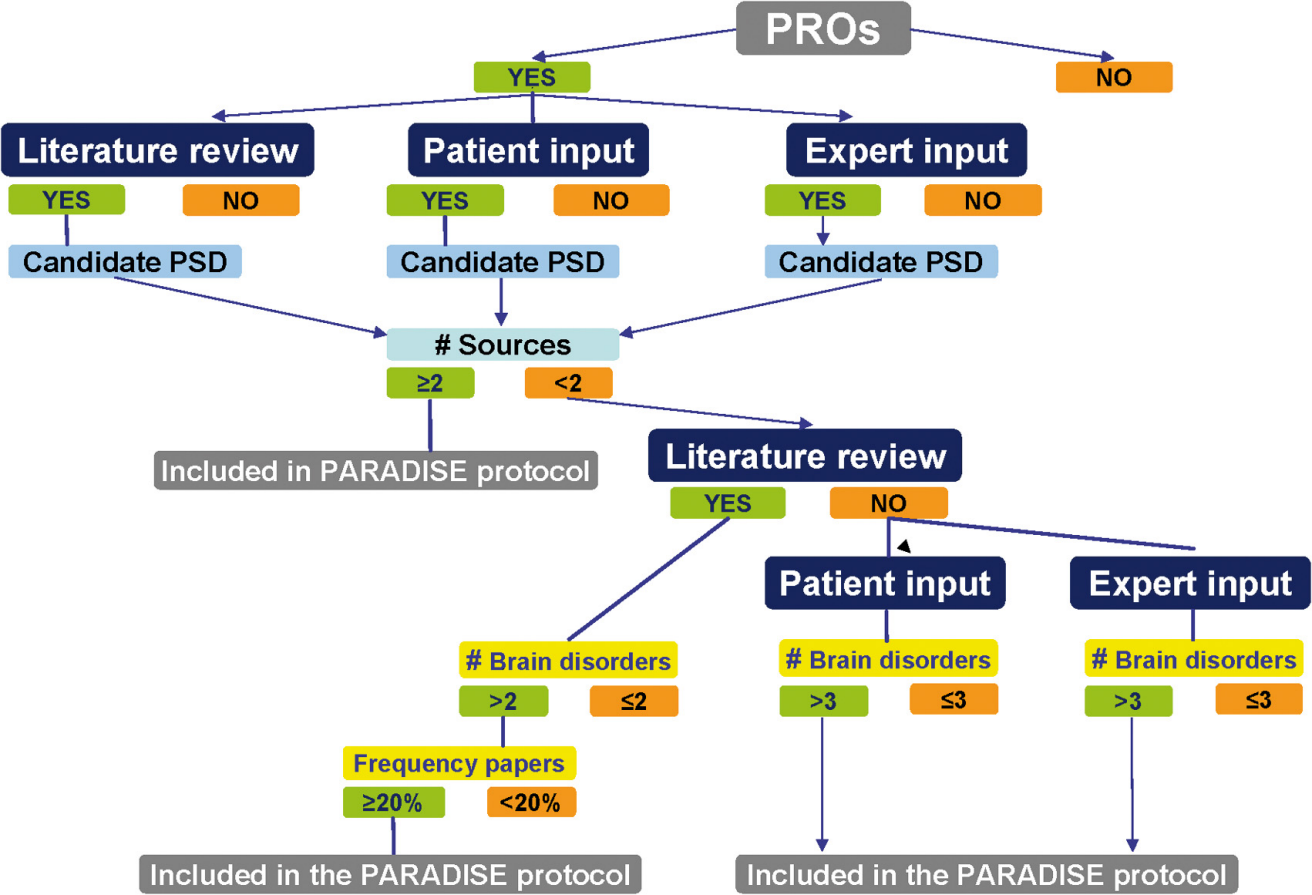
Decision tree used for selecting relevant psychosocial difficulties across brain disorders. The starting point of the decision tree was the list of psychosocial difficulties (PSD) addressed in the 27 patient reported outcome (PRO) or outcome instruments identified in the literature reviews. If a PSD was addressed in at least one of the PROs or outcome instruments and had also been identified in at least two of the three sources of information (literature reviews, qualitative study and clinical input), this PSD was selected for inclusion into the data collection protocol. If a PSD had only been included in one of the sources of information, then if it had been identified in the literature reviews of at least two brain disorders and in those in > 20% of the studies included in the reviews, then the PSD was also included in the PARADISE protocol. If not, then if it had been included in the patient input studies for more than three brain disorders, or in the clinical input for more than three brain disorders, in both cases it was included in the protocol.

Potentially relevant PSDs and determinants had to be selected for inclusion in the data collection protocol. This was done separately for PSDs and determinants. Since we wanted to test the hypothesis of horizontal epidemiology we had to make sure that even PSDs and determinants relevant to small group of disorders were included. At the same time we had to keep the balance between comprehensiveness and practicability. The procedure for selecting PSDs is shown in Fig 2. We included in the data collection protocol those determinants identified in at least two of the literature reviews or in at least two brain disorders in the qualitative study.

The selection of PSDs and determinants was critically evaluated by a panel of six external experts. The members of this independent panel were selected based on their outstanding expertise as clinicians and researchers and for holding leadership positions in Europe in the field of brain disorders. The experts were asked to provide additional PSDs and determinants they thought were missing in the protocol based on their experience of patients with the selected brain disorders.

### (6) Construction and piloting of the data collection protocol

For most of the PSDs and determinants, questions from questionnaires and clinical instruments and national and international health surveys were identified and used for the data collection protocol. Instruments used were: WHO World Health Survey (http://www.who.int/healthinfo/survey/en/); WHO Composite International Diagnostic Interview (CIDI) [20]; WHO Disability Assessment Schedule 2.0 (WHODAS II) [21]; Hospital Anxiety and Depression Scale (HADS) [22]; Quality of Life Instrument for Young Hemorrhagic Stroke Patients (HSQuale) [23]; Sickness Impact Profile (SIP) [24]; Symptom Checklist (SCL-90) [25]; Schedule for Clinical Assessment in Neuropsychiatry (SCAN) [26]; Stroke Adapted-Sickness Impact Profile (SA-SIP) [27]; Stroke Impact Scale (SIS) [28]; WHO Survey on Health and Health System Responsiveness (HSQR) (http://www.who.int/responsiveness/surveys/en/); WHO Quality of Life (WHOQoL) [29]. In a few cases, where no standard question was available, new questions were developed. The response options were homogenized to be the same for all PSDs questions, namely None, Mild, Moderate, Severe, Extreme, Don’t know and not applicable. The response options for environmental determinants ranged from No positive influence to Strong positive influence and from No negative influence to Strong negative influence. The determinants referring to personal characteristics, such as personality or self-efficacy, were collected with standard questionnaires, such as the 10-item short version of the Big Five Inventory [30] and items of the General Self-Efficacy Scale [31]. For those instruments the original questions and response options were kept. The data from these questionnaires were not analyzed for this paper.

The protocol also contained a section in which participants were asked 1) whether there are problems or difficulties not mentioned during the interview but which they would like to add and 2) to mention up to five problems or difficulties in areas of life that are the most troubling or burdensome for them. For these, the participants were also asked when it was that they first occurred, how they had changed over time, and whether something influenced their change.

The feasibility and clarity of the protocol were piloted in interviews with four patients, two with depression, one with schizophrenia and one with Parkinson’s disease.

### (7) Implementation of the data collection protocol

The data collection protocol was implemented in a cross-sectional study on a convenience sample of 80 persons per brain disorder. Patients were interviewed by a trained clinical researcher using the protocol. For the purpose of describing the study population, demographic information, age, the impact of comorbidities and a general health question were asked. Comorbidities were accessed with the Self-reported Comorbidities Questionnaire (SCQ) [32]. Using the SCQ a summary score on the impact of comorbidity is derived by adding the up to three points obtained from each of the included 12 health conditions: one point for its presence, one if treatment is received, and one if it causes decrements in functioning. In addition, to determine the severity of the brain disorder, standard disease-specific measures routinely used in the recruiting centres, were used. In the case of epilepsy, no instrument was routinely used, so severity of epilepsy was assessed by frequency of seizures in the last year and the number of anti-epileptic drugs taken.

Patient inclusion criteria were that the main diagnosis was one of the nine brain disorders and the patient was at least 18 years old with sufficient facility with the language of the country to understand the purposes of consent. The patient was asked to agree to participate and to sign the informed consent form. For dementia patients not able to provide consent, the caregivers were asked to give consent and to participate in the interviews as proxies.

The data of the patients with dementia and schizophrenia were collected at the Instytut Psychiatrii i Neurologii in Warsaw, Poland, the data of those with epilepsy, migraine, multiple sclerosis, Parkinson´s disease and stroke at the Instituto Nazionale Neurologico “Carlo Besta” in Milan, Italy, of those with substance dependency at the Järvanpää Addiction Hospital, Haarajoki, Finland and of those with depression at the the Hospital Universitario de La Princesa in Madrid, Spain.

### Testing the hypothesis of ‘horizontal epidemiology’

It was previously decided that a PSD was associated with a brain disorder if at least 25% of patients reported it as a difficulty, independently of the severity (Mild, Moderate, Severe or Extreme). It was also decided that a PSD could be said to be commonly experienced across brain disorders if 1) the PSD was associated to 5 out of the 9 brain disorders and 2) among the 5 both neurological conditions (epilepsy, migraine, multiple sclerosis, Parkinson’s disease, stroke) and psychiatric conditions (dementia, depression, schizophrenia, substance dependency) were represented. Even though it could be questioned whether dementia is a neurological or a psychiatric condition, we followed the ICD-10 and classified it as psychiatric. The same criteria were used for testing whether there are environmental determinants with cross-cutting influence, either positive or negative. For the calculation of the percentages the response option don’t know and not applicable were considered missing data.

To describe our sample we also used descriptive statistics. The severity of the brain disorder was determined by dividing each patient group into categories of mild, moderate and severe according to standardized cut-offs found in the literature for the corresponding instruments.

## Results

The multi-method approach used to identify potentially relevant PSDs and their determinants across brain disorders resulted in the so called PARADISE data collection protocol, which includes 64 PSDs and 59 determinants of PSD. Columns one to three of Table 1 present the PSDs included in the protocol by name, by ICF code, and the question used to operationalize them. Columns 4 to 12 of Table 1 summarize the results of the cross-sectional study and present for each PSD and each brain disorder, the percentage of people who had reported difficulties. Those cells above 25% are marked in bold. Table 2 shows demographic characteristics as well as the allocation to severity group of the 722 people participating in the cross-sectional study.

**Table 1:** Psychosocial difficulties (PSD) included in the PARADISE data collection protocol.

Psychosocial difficulties^§^			Epilepsy	Migraine	Multiple Sclerosis	Parkinson	Stroke	Dementia	Depression	Schizophrenia	Substance dependency
ICF Code	PSD Name	Operationalization: Question (Source*)	Percentage of persons reporting difficulty								
**Mental functions**											
b130	Energy and drive functions	How much of a problem did you have due to not feeling rested and refreshed during the day (e.g. feeling tired, not having energy)? (WHS)	**80.0**	**95.0**	**81.3**	**75.0**	**71.3**	**83.8**	**92.6**	**86.4**	**92.5**
b1301	Motivation	How much of a problem did you have not finding things that kept you interested and motivated?	**45.0**	**57.5**	**48.8**	**30.0**	**45.0**	**67.5**	**91.4**	**79.0**	**87.5**
b1302	Appetite	How much of a problem did you have with your appetite?	**26.3**	**41.3**	20.0	16.3	15.0	**40.0**	**63.0**	**50.6**	**51.3**
b1304	Impulse control	How much of a problem did you have resisting doing or saying things in ways you would normally think were inappropriate? (CIDI)	18.8	**52.5**	**38.8**	**30.0**	15.0	**37.5**	**56.8**	**60.5**	**60.0**
b134	Sleep functions	How much of a problem did you have with sleeping, such as falling asleep, waking up frequently during the night or waking up too early in the morning? (WHS)	**46.3**	**71.3**	**57.5**	**70.0**	**46.3**	**57.5**	**86.4**	**72.8**	**83.8**
b140	Attention functions	How much difficulty did you have in concentrating on doing something for ten minutes? (WHODAS II)	**36.3**	**62.5**	**30.0**	**38.8**	**50.0**	**41.3**	**77.8**	**56.8**	**68.8**
b144	Memory functions	How much difficulty did you have in remembering to do important things? (WHODAS II)	**57.5**	**47.5**	**36.3**	**32.5**	**62.5**	**91.3**	**77.8**	**65.4**	**82.5**
b147	Psychomotor functions	How much of a problem did you have with being slowed down or feeling as if things were moving too fast around you? (Based on HADS)	**33.8**	**66.3**	**50.0**	**81.3**	**65.0**	**72.5**	**80.3**	**67.9**	**65.0**
b147	Agitation & Aggression / Hyperactivity	How much of a problem did you have being so irritable that you started arguments, shouted at people or even hit people? (Based on CIDI)	20.0	**55.0**	**41.3**	**36.3**	25.0	**53.8**	**75.3**	**44.4**	**71.3**
b152	Emotional functions	How much have you been emotionally affected by your < health condition >? (WHODAS II)	**73.8**	**88.8**	**78.8**	**66.3**	**80.0**	**67.5**	**97.5**	**77.8**	**90.0**
b152	Depressive mood	How much of a problem did you have with feeling sad, low or depressed? (WHS)	**66.3**	**81.3**	**61.3**	**63.8**	**71.3**	**76.3**	**98.8**	**81.5**	**88.8**
b152	Worry and anxiety	How much of a problem did you have with worry or anxiety? (WHS)	**68.8**	**87.5**	**73.8**	**61.3**	**72.5**	**65.0**	**96.3**	**80.3**	**92.5**
b152	Stress	How much of a problem did you have with not being able to cope with all the things that you had to do? (WHS)	**55.0**	**81.3**	**55.0**	**70.0**	**57.5**	**67.5**	**77.8**	**67.9**	**76.3**
b1521	Regulation of emotions	How much of a problem did you have with emotional or mood swings, such as laughing or crying suddenly for no reason? (Based on HSQuale and SIP)	**35.0**	**48.8**	**37.5**	**42.5**	**52.5**	**41.3**	**66.7**	**58.0**	**87.5**
b156	Perceptual functions / Sensoperceptive functions	How much of a problem did you have with hearing or seeing things that other people do not hear or see?	6.3	15.0	8.8	11.3	3.8	15.0	12.4	**45.7**	23.8
b152	Feeling empty, emptiness / loneliness	How much of a problem did you have with feeling “empty” inside? (Based on CIDI)	17.5	**46.3**	**32.5**	**31.3**	17.5	**33.8**	**84.0**	**63.0**	**81.3**
b152	Feeling empty, emptiness / loneliness	How much of a problem did you have with feeling lonely even when with people? (Based on SCL-90)	22.5	**38.8**	**40.0**	25.0	22.5	**48.8**	**79.0**	**82.7**	**77.5**
B1678	Linguistic processing	How much of a problem did you have in finding the words you wanted to say or understanding words said to you? (Based on HSQuale)	**40.0**	**62.5**	**38.8**	**63.8**	**50.0**	**66.3**	**76.5**	**74.1**	**77.5**
b160	Thought functions	How much of a problem did you have with feeling that your thoughts were too slow or that you could not think clearly? (SCAN)	**28.8**	**57.5**	**31.3**	**27.5**	**48.8**	**48.8**	**79.0**	**71.6**	**77.5**
b1602	Irrational fears / phobias	How much of a problem did you have with feeling strong fear even though you knew there was no real danger? (Based on CIDI)	13.8	**35.0**	**26.3**	18.8	17.5	**27.5**	**51.9**	**61.7**	**58.8**
b164	Executive functions	How much difficulty did you have in making decisions?	25.0	**48.8**	**36.3**	**40.0**	**28.8**	**67.5**	**74.1**	**70.4**	**78.8**
b164	Executive functions	How much difficulty did you have in analysing and finding solutions to problems in day-to-day life? (WHODAS II)	22.5	**43.8**	22.5	**36.3**	**30.0**	**71.3**	**72.8**	**67.9**	**81.3**
**Other body functions under central neurological control**											
b2	Sensory functions	How much of a problem did you have with sensory disturbances, such as hypersensitivity to light or noise, numbness, tingling, blurred or double vision?	**26,3**	**75,0**	**56,3**	**62,5**	**40,0**	25,0	**59,3**	**64,2**	**60,0**
b280	Pain	How much bodily ache or pain did you have? (WHS)	**48,8**	**93,8**	**48,8**	**53,8**	**51,3**	**68,8**	**82,7**	**59,3**	**81,3**
b310, b320	Speaking	How much of a problem did you have in speaking clearly? (Based on SA-SIP)	**32.5**	**45.0**	25.0	**47.5**	**51.3**	15.0	**50.6**	**46.9**	**55.0**
b525	Bowel functions	How much of a problem did you have with controlling your bowels, such as being constipated or having diarrhoea? (Based on SIS/HAQUAMS)	25.0	**48.8**	**50.0**	**46.3**	**28.8**	**41.3**	**60.5**	**44.4**	**65.0**
b530	Weight maintenance functions	How much of a problem did you have with gaining or losing weight?	21.3	**35.0**	**37.5**	**38.8**	17.5	**30.0**	**58.0**	**59.3**	**57.5**
b620	Urination functions	How much of a problem did you have with passing water (urinating) or in controlling urine (incontinence)? (HSQR)	5.0	11.3	**40.0**	**38.8**	21.3	**36.3**	24.7	**33.3**	**31.3**
b640	Sexual functions	How much difficulty did you have in sexual activities? (WHODAS II)	23.8	23.8	18.8	**28.8**	21.3	7.5	**65.4**	**27.2**	**38.8**
	Libido	How much of a problem did you have with your level of sexual desire?	16.3	**31.3**	**32.5**	**42.5**	17.5	7.5	**81.5**	**39.5**	**51.3**
b730	Muscle power functions	How much of a problem did you have with the strength of your muscles in your face, arms or legs?	17.5	**46.3**	**56.3**	**56.3**	**60.0**	**27.5**	**55.6**	**40.7**	**58.8**
b735	Muscle tone functions	How much of a problem did you have with increased muscle tone or stiffness?	13.8	22.5	**38.8**	**71.3**	**45.0**	18.8	**48.2**	**48.2**	**66.3**
b760	Control of voluntary movement functions, coordination, gait	How much of a problem did you have with coordinating your movements?	17.5	22.5	**47.5**	**71.3**	**60.0**	**45.0**	**45.7**	**45.7**	**61.3**
b7	Balance	How much of a problem did you have with your balance?	18.8	**37.5**	**60.0**	**66.3**	**58.8**	**53.8**	**51.9**	**38.3**	**58.8**
b765	Involuntary movement functions	How much of a problem did you have with tremor (e.g. shaking and trembling) in your arms or legs?	21.3	**26.3**	22.5	**68.8**	**31.3**	23.8	**45.7**	**48.2**	**61.3**
**Difficulties in activities and participation**											
d1	Learning and applying knowledge	How much difficulty did you have in learning a new task, for example, learning how to get to a new place? (WHODAS II)	15.0	**47.5**	21.3	**31.3**	**31.3**	**80.0**	**50.6**	**56.8**	**65.0**
d166	Reading	How much difficulty did you have in reading books, instructions or newspapers?	16.3	**46.3**	25.0	25.0	**31.3**	**66.3**	**70.4**	**71.6**	**56.3**
d230	Carrying out daily routine	How much difficulty did you have in carrying out your day-to-day activities?	**42.5**	**68.8**	**46.3**	**45.0**	**61.3**	**72.5**	**87.7**	**66.7**	**57.5**
d3	Communication	How much difficulty did you have in generally understanding what people say? (WHODAS II)	11.3	**36.3**	6.3	16.3	18.8	**61.3**	**35.8**	**64.2**	**52.5**
d3	Communication	How much difficulty did you have in starting and maintaining a conversation? (WHODAS II)	13.8	**36.3**	12.5	**31.3**	**31.3**	**67.5**	**50.6**	**65.4**	**65.0**
d4	Mobility (in general)	How much difficulty did you have in standing for long periods such as 30 minutes? (WHODAS II)	12.5	**40.0**	**42.5**	**60.0**	**48.8**	**38.8**	**63.0**	**61.7**	**62.5**
d430	Lifting and carrying objects	How much difficulty did you have in lifting and carrying things?	12.5	**47.5**	**48.8**	**56.3**	**43.8**	**75.0**	**66.7**	**56.8**	**51.3**
d440 d445	Hand and arm use and fine hand use	How much difficulty did you have in using your hands and fingers, such as picking up small objects or opening or closing containers? (HSQR)	5.0	13.8	**38.8**	**65.0**	**32.5**	**31.3**	**44.4**	**27.2**	**38.8**
d455	Moving around	How much difficulty did you have with moving around? (WHS)	5.0	17.5	**27.5**	**38.8**	**40.0**	**46.3**	**37.0**	**40.7**	**40.0**
d450	Walking	How much difficulty did you have in walking a long distance such as a kilometre (or equivalent)? (WHODAS II)	17.5	**43.8**	**41.3**	**56.3**	**51.3**	**65.0**	**48.2**	**50.6**	**46.3**
d475	Driving	How much difficulty did you have in driving?	**26.3**	**27.5**	23.8	**37.5**	**43.8**	13.8	**32.1**	13.6	8.8
d510	Washing oneself	How much difficulty did you have in washing your whole body? (WHODAS II)	3.8	7.5	15.0	22.5	23.8	**38.8**	**32.1**	**35.8**	**27.5**
d530	Toileting	How much difficulty did you have in using the toilet?	5.0	17.5	10.0	16.3	11.3	23.8	19.8	16.1	8.8
d540	Dressing	How much difficulty did you have in getting dressed? (WHODAS II)	5.0	7.5	13.8	**43.8**	**27.5**	**28.8**	**32.1**	21.0	22.5
d550	Eating	How much difficulty did you have in eating? (WHODAS II)	1.3	16.3	5.0	16.3	11.3	13.8	**35.8**	14.8	16.3
d5	Independency in everyday activities	How much difficulty did you have in staying by yourself for a few days? (WHODAS II)	10.0	11.3	11.3	12.5	**31.3**	**56.3**	**45.7**	**49.4**	**60.0**
d570	Looking after one’s health	How much difficulty did you have with looking after your health, such as eating well, exercising and taking your medicines?	5.0	**30.0**	23.8	17.5	18.8	**71.3**	**48.2**	**60.5**	**66.3**
d640	Doing housework	How much difficulty did you have in taking care of your household responsibilities? (WHODAS II)	18.8	**48.8**	**32.5**	25.0	**47.5**	**77.5**	**79.0**	**64.2**	**66.3**
d660	Caring for others	How much difficulty did you have in providing for or supporting others? (Based on WHOQoL)	15.0	**42.5**	25.0	23.8	**26.3**	**57.5**	**39.5**	**51.9**	**65.0**
d7	Interpersonal interactions and relationships	How much difficulty did you have in dealing with conflicts and tensions with others? (WHS)	15.0	**50.0**	**35.0**	**28.8**	16.3	**50.0**	**67.9**	**70.4**	**77.5**
d7	Interpersonal interactions and relationships	How much difficulty did you have in dealing with people you do not know? (WHODAS II)	16.3	**37.5**	22.5	**32.5**	21.3	**57.5**	**32.1**	**69.1**	**68.8**
d7500	Informal relationships with friends	How much difficulty did you have in maintaining a friendship? (WHODAS II)	22.5	**36.3**	12.5	16.3	22.5	**47.5**	**44.4**	**61.7**	**68.8**
d760 + d770	Family relationships and intimate relationships	How much difficulty did you have in getting along with people who are close to you? (WHODAS II)	10.0	**45.0**	20.0	18.8	**28.8**	**48.8**	**35.8**	**65.4**	**58.8**
d839 + d850	Education / Work and employment	How much difficulty did you have in your day-to-day work or school? (WHODAS II)	**42.5**	**55.0**	**38.8**	22.5	**26.3**	25.0	**48.2**	**35.8**	22.5
d870	Economic self-sufficiency	How much difficulty did you have with having enough money to meet your needs? (Based on the WHOQoL)	22.5	**38.8**	**41.3**	**26.3**	**33.8**	**38.8**	**40.7**	**70.4**	**85.0**
d870	Economic self-sufficiency	How much difficulty did you have with managing your money?	15.0	**30.0**	**26.3**	17.5	17.5	**50.0**	22.2	**64.2**	**85.0**
d9	Community, social and civic life	How much difficulty did you have in joining in community activities (for example, festivities, religious or other activities) in the same way as anyone else can? (WHODAS II)	16.3	**51.3**	**32.5**	**28.8**	**28.8**	**51.3**	**69.1**	**60.5**	**85.0**
d920	Recreation and leisure	How much difficulty did you have in doing things by yourself for relaxation or pleasure? (WHODAS II)	16.3	**40.0**	**26.3**	13.8	**28.8**	**53.8**	**75.3**	**58.0**	**80.0**

^**§**^ Columns one to three present all PSDs indicating the code of the ICF classification they address and the question we used to operationalize them. Columns 4 to 12 present for each PSD and each brain disorder, the percentage of persons, who had reported difficulties in the study implementing the PARADISE data collection protocol. Those cells above 25% are marked in bold.

*WHS: World Health Organization (WHO) World Health Survey; CIDI: WHO Composite International Diagnostic Interview; WHODAS II: WHO Disability Assessment Schedule 2.0; HADS: Hospital Anxiety and Depression Scale; HSQuale: Quality of Life Instrument for Young Hemorrhagic Stroke Patients; SIP: Sickness Impact Profile; SCL-90: Symptom Checklist; SCAN: Schedule for Clinical Assessment in Neuropsychiatry; SA-SIP: Stroke Adapted-Sickness Impact Profile; SIS: Stroke Impact Scale; HSQR: WHO Survey on Health and Health System Responsiveness; WHOQoL: WHO Quality of Life.

**Table 2:** Demographic characteristics of the persons participating in the study implementing the PARADISE data collection protocol.

		Epilepsy	Migraine	Multiple Sclerosis	Parkinson	Stroke	Dementia	Depression	Schizophrenia	Substance dependency
**N**		80	80	80	80	80	80	81	81	80
**Country (data collection)** ^**§**^		Italy	Italy	Italy	Italy	Italy	Poland	Spain	Poland	Finland
**Age (years)**	**Mean**	41,23	44,54	41,03	61,24	59,84	81,03	54,81	38,38	39,56
	**SD**	11,99	12,12	8,74	10,45	14,36	5,49	14,73	14,03	13,15
**Gender (%)**	**Female**	50,0%	86,3%	65,0%	40,0%	43,8%	78,8%	82,7%	53,1%	37,5%
**Setting (N)**	**Inpatient**	28	0	9	24	79	10	3	56	80
	**Outpatient care**	31	80	70	56	1	68	77	9	0
	**Home care**	21	0	1	0	0	1	0	1	0
	**Other**	-	-	-	-	-	1	1	15	-
**General living situation (%)**	**Living independently and alone**	11,3%	12,5%	15,0%	13,8%	10,0%	25,0%	34,6%	23,5%	41,3%
	**Living independently with others in a household**	88,8%	87,5%	83,8%	86,3%	83,8%	55,0%	59,3%	65,4%	42,5%
**Marital status (N)**	**Married**	36	42	45	61	57	25	32	6	16
	**Marriage-like relationship**	6	11	5	4	5	0	5	4	13
	**Separated**	3	5	4	2	3	0	4	0	1
	**Divorced**	2	2	1	1	0	2	10	8	10
	**Widowed**	1	3	2	5	2	48	13	3	2
	**Never married**	32	17	23	7	13	5	17	60	38
**Highest level of education completed (%)**	**No formal schooling**	0,0%	0,0%	1,3%	0,0%	0,0%	0,0%	2,5%	0,0%	0,0%
	**Less than primary school**	0,0%	0,0%	0,0%	1,3%	0,0%	3,8%	14,8%	0,0%	3,8%
	**Primary school completed**	1,3%	1,3%	1,3%	13,8%	22,5%	18,8%	16,0%	4,9%	35,0%
	**Secondary school completed**	28,8%	18,8%	23,8%	26,3%	22,5%	3,8%	11,1%	4,9%	32,5%
	**High school completed**	50,0%	48,8%	51,3%	43,8%	40,0%	40,0%	17,3%	44,4%	17,5%
	**University completed**	20,0%	28,8%	20,0%	13,8%	12,5%	27,5%	27,2%	43,2%	6,3%
	**Post graduate degree completed**	0,0%	2,5%	2,5%	1,3%	2,5%	6,3%	11,1%	2,5%	5,0%
**Working sample (%)**	** **	66,3%	67,5%	72,5%	33,8%	25,0%	0,0%	27,2%	8,6%	6,3%
**Disease duration (years)**	**Mean**	18,67	21,13	7,66	6,26	4,00	3,69	12,63	13,03	12,16
	**SD**	12,32	14,60	6,94	4,40	6,48	2,70	11,57	11,83	8,67
**Disease severity**	**Instrument** *	**CRS**	**MIDAS**	**EDSS**	**Hoehn & Yahr**	**NIHSS**	**MMSE**	**HDRS**	**CGI**	**ADS** **
	**N**	79	80	80	80	55	80	81	81	34
	**Mean (SD)**	na	27,16 (22,92)	2,13(1,74)	na	4,93(4,39)	21,10 (2,89)	19,70 (5,49)	na	
	**Cut-off Mild Severity**	**= 1**	**<6**	**<3**	**= 1 or 1.5**	**1 to 5**	**≥25**	**<14**	**= 2 or 3**	**≤ 13**
	**No of persons**	24	9	64	15	37	6	11	32	1
	**Cut-off Moderate Severity**	**= 2**	**≥ 6 & ≤ 20**	**≥ 3 & ≤ 5**	**= 2 or 2.5**	**6 to 14**	**≥ 10 & < 25**	**≥ 14 & ≤ 18**	**= 4 or 5**	**≥ 14 & ≤ 21**
	**No of persons**	28	27	9	58	14	74	26	49	7
	**Cut-off High Severity**	**= 3**	**>20**	**> 5**	**= 3 or 4**	**≥ 15**	**< 10**	**>18**	**= 6 or 7**	**≥ 22**
	**No of persons**	27	44	7	7	29	0	44	0	26
**Comorbidity score (SCQ score** *** **)**	**Mean**	2,36	2,63	1,18	3,00	5,87	5,86	12,56	2,72	8,84
	**SD**	2,97	2,55	2,17	2,63	4,87	4,17	5,09	3,14	4,76
**In general, how would you rate your health today? (%)**	**Very good**	11,3%	3,8%	15,0%	3,8%	2,5%	3,8%	2,5%	11,1%	1,3%
	**Good**	43,8%	47,5%	43,8%	51,3%	37,5%	26,3%	18,5%	37,0%	38,8%
	**Neither poor nor good**	35,0%	36,3%	32,5%	41,3%	41,3%	57,5%	24,7%	42,0%	41,3%
	**Poor**	6,3%	10,0%	7,5%	3,8%	13,8%	11,3%	39,5%	9,9%	17,5%
	**Very poor**	3,8%	2,5%	1,3%	0,0%	5,0%	1,3%	14,8%	0,0%	1,3%

^§^ Data collection centers: dementia and schizophrenia, Instytut Psychiatrii i Neurologii in Warsaw, Poland; epilepsy, migraine, multiple sclerosis, Parkinson´s disease and stroke, Instituto Nazionale Neurologico “Carlo Besta” in Milan, Italy; substance dependency, Järvanpää Addiction Hospital, Haarajoki, Finland and depression, Hospital Universitario de La Princesa in Madrid, Spain.

* HDRS: Hamilton Depression Rating Scale; CRS: Clinical Rating of Severity; MIDAS: Migraine Disability Assessment; EDSS: Expanded Disability Status Scale; Hoehn & Yahr: Hoehn & Yahr Score; NIHSS: National Institutes of Health Stroke Scale; CGI: Clinical Global Impression (CGI); MMSE: Mini Mental State Examination; ADS: Alcohol Dependence Scale.

** In substance dependency, 44 persons had alcohol dependence as their main diagnosis. The data reported here refer to the 34 of those from whom the ADS data were available. Mean is not reported because of the low N. For all other substance dependency conditions, the intention was to collect data with the ‘Severity of Dependence Scale’. There were, however, a larger number missing data and the results are, therefore, not reported.

*** SCQ Score: Self-reported Comorbidities Questionnaire. The summary score is derived by adding the up to three points obtained from each reported health conditions: one point for its presence, one if treatment is received, and one if it causes decrements in functioning

In 57 of the 64 PSDs more than 25% of the sample of at least 5 brain disorders reported difficulties. In all 57 both neurological and psychiatric brain disorders were represented. Problems with mental functions like being emotionally affected, depressive mood, worry and anxiety, stress and pain were, however, meaningfully more frequently than our 25% cut-off: these PSDs were endorsed by more than 45% of all persons with brain disorders independently of the underlying condition. Problems with the level of energy and tiredness were even more frequent and endorsed for all included brain disorders by more than 70% of the different samples. Regarding activities and participation domains, problems in carrying out daily routine were endorsed by more than 40% of the persons with any condition, followed by problems in mobility, lifting and carrying objects, walking and economic sufficiency, endorsed by more than ca. 40% of all subsamples but epilepsy and PD.

Table 3 presents environmental determinants with percentage of people who felt that these determinants influenced their PSDs, either positively or negatively. Seventeen of the 20 environmental determinants fulfil our criteria as common influences of PSDs. Extremely important determinants across conditions were both care and attitudes of health professionals, and help and assistance as well as attitudes of the family, endorsed by more than 80% and 70% of the sample regardless of brain disorder, respectively. More than 84% of persons with any condition endorsed the impact of medication in their PSD, with the exception of substance dependency (50% endorsement rate). The four environmental determinants not fulfilling our criteria were ‘costs of the medication’, ‘assistive devices’, ‘access to alcohol’ and ‘access to illegal drugs’. As can be seen from Table 3, in general terms the percentages of people endorsing the influence of environmental determinants on their PSD were very high in all groups.

**Table 3:** Environmental determinants included in the data collection protocol and the percentage of persons who experienced them as having a positive or negative influence in their psychosocial difficulties.

Determinant	Epilepsy	Migraine	Multiple Sclerosis	Parkinson	Stroke	Dementia	Depression	Schizophrenia	Substance dependency
Medication	**98.8**	**90.0**	**91.3**	**93.8**	**92.5**	**86.3**	**84.0**	**92.6**	**50.0**
Costs of the medication	18.8	**48.8**	**68.8**	10.0	13.8	**60.0**	19.8	**37.0**	17.5
Other treatments	**28.8**	**30.0**	**42.5**	**35.0**	**46.3**	**31.3**	**54.3**	**61.7**	**96.3**
The environment around you	**30.0**	**57.5**	**57.5**	**48.8**	18.8	**96.3**	**50.6**	**42.0**	**58.8**
Public transportation	**50.0**	**55.0**	**43.8**	**30.0**	22.5	**50.0**	**45.7**	**46.9**	**48.8**
Assistive devices	22.5	13.8	16.3	16.3	**65.0**	25.0	**34.6**	2.5	5.0
Weather or climate	21.3	**90.0**	**77.5**	**67.5**	15.0	**68.8**	**72.8**	**77.8**	**61.3**
Awareness of health condition people have	**58.8**	**75.0**	**67.5**	**48.8**	**35.0**	**61.3**	**63.0**	**74.1**	**83.8**
Help and assistance from family	**98.8**	**86.3**	**87.5**	**81.3**	**98.8**	**97.5**	**81.5**	**90.1**	**95.0**
Attitudes of family	**96.3**	**88.8**	**87.5**	**92.5**	**98.8**	**95.0**	**69.1**	**81.5**	**83.8**
Help and assistance from friends	**80.0**	**66.3**	**80.0**	**38.8**	**66.3**	**57.5**	**72.8**	**63.0**	**76.3**
Attitudes of friends	**80.0**	**67.5**	**81.3**	**75.0**	**71.3**	**66.3**	**59.3**	**63.0**	**76.3**
Help and assistance of peers or colleagues	**67.5**	**56.3**	**57.5**	22.5	**46.3**	**41.3**	**33.3**	**61.7**	**81.3**
Attitudes of peers and colleagues	**77.5**	**61.3**	**63.8**	**48.8**	**50.0**	**53.8**	**35.8**	**60.5**	**78.8**
Attitudes of strangers	**41.3**	**30.0**	**37.5**	**27.5**	15.0	**62.5**	**43.2**	**63.0**	**36.3**
Care from health professionals	**100.0**	**83.8**	**82.5**	**95.0**	**97.5**	**90.0**	**90.1**	**91.4**	**96.3**
Attitudes of health professionals	**93.8**	**83.8**	**86.3**	**93.8**	**97.5**	**83.8**	**80.3**	**85.2**	**86.3**
Health problems of other members of family	23.8	**65.0**	**46.3**	22.5	21.3	**67.5**	**70.4**	**66.7**	**50.0**
Access to alcohol	2.5	7.5	10.0	2.5	1.3	1.3	9.9	13.6	**58.8**
Access to illegal drugs	0.0	0.0	6.3	0.0	0.0	0.0	2.5	6.2	**51.3**

## Discussion

The hypothesis of horizontal epidemiology in brain disorders, namely, that there are PSDs and determinants experienced in common across brain disorders was supported by the evidence in this paper. This finding challenges the brain disorder-specific, silo-like, or vertical approach in which clinical and epidemiological research about psychosocial difficulties is commonly carried out and corresponding health care delivery practiced in many countries of the world.

To our knowledge this is the first time that this hypothesis has been formulated and tested by means of an approach integrating information from literature, PROs and clinical instruments, expert clinical knowledge and most importantly the involvement of persons with brain disorders themselves. Results of the steps that led to the development of the PARADISE data collection protocol and determinants have been recently published [14, 16–18, 33–35]. Here we concentrate exclusively on the results and discussion of the testing of the hypothesis of horizontal epidemiology.

We were surprised by the result that 56 of 64 PSDs fulfilled the criteria we established to test the hypothesis of horizontal epidemiology and that for several PSD much larger rates of endorsement across brain conditions were observed. As expected due to the overlap with symptoms of mental and neurological disorders, PSDs related to mental health functions, especially being emotionally affected, depressive mood, worry and anxiety, and coping with stress were highly relevant and affected nearly half of our participants independently of their brain disorder. The same pattern was observed for pain, with extremely high endorsement rates in persons with migraine (94%) but also in persons with depression and substance dependency (over 80%). Further highly relevant PSD across disorders were problems in carrying out daily routine, in mobility and in keeping economic sufficiency. These results confirm, first of all, the impact of brain disorders on people’s lives. This impact goes beyond impairments, such as sleep disturbances, to fundamental difficulties in major life areas, such as keeping economic sufficiency. Similar results have already been shown for single conditions such as migraine, PD, MS, stroke, Dementia, depression or epilepsy [8,17–22]. Similarities regarding PSD have also been shown in studies across mental disorders applying the WHO Disability Assessment Schedule (WHODAS 2.0), a generic instrument for health and disability valid across diseases and disorders, also mental disorders [21]. Exemplary articles report that problems are experienced to different extents in all six WHODAS domains (cognition, mobility, self-care, getting along with others, participation in society, and life activities) by persons with any mood, anxiety or substance use disorder [36] or persons with bipolar affective disorder and schizophrenia [37]. The novelty of our work consists of showing that people experience the same kind of difficulties regardless of the brain disorder they have, in a broad range of brain disorders specific PSD selected in a multi-step process and in altogether nine brain disorders very different regarding aetiology, symptoms and progression.

Similarly surprising clear results were observed for environmental determinants: 16 from 20 determinants fulfilled the criteria we established a priori, and the endorsement rates for these determinants were generally much higher than the 25% criteria, regardless of brain condition. Extremely high endorsement rates across brain disorders for care and attitudes of health professionals, and help and assistance as well as attitudes of the family stress the importance of these relationships for PSD. These results are in line with the acknowledged importance of family support in different brain disorders, for instance in PD and Dementia [38], and in line with current efforts to improve service user experience in adult mental health, where one quality statement is assuring that users “*and their families or carers*, *are treated with empathy*, *dignity and respect*” [12]. Again, the novelty of our work consists rather of showing that these determinants are valid across brain disorders.

Our results were unexpected because we included a large number of PSDs and determinants in the data collection protocol so as not to miss potentially relevant PSDs and determinants. One may object that the result was affected by the overly lenient cut-off of 25% for relevant PSD and 5 out of 9 brain disorders. But, one out of four is already highly significant, and we had no reason to set that threshold higher. In the case of using 5 out of 9 brain disorders as the threshold for commonality, we feel very confident that this was sufficient since the results would have been unchanged if a higher cut-off 7 out of 9 brain disorders was used.

One might object as well that a major limitation of our study was the lack of a comparison of PSDs experienced by persons with disorders that cause chronic ill health, other than brain disorders. It is certainly our hypothesis that many of the PSDs shared by brain conditions would be shown to be experienced by persons with other chronic health conditions, even those that are not brain disorders. A recent study to create a 'generic ICF core set' found that for a large clinical population (N = 9863) with very diverse health conditions, several functioning domains–mobility, self-care and interpersonal relationships, among others–were experienced in common and explained their general health status [39]. Many of these categories were indeed the same as the PSDs reported in this study. At the same time, for some health conditions such as asthma, or disease groups, such as lung diseases, it is likely that there are fairly specific PSD that are highly relevant both to research and treatment, but might not be common problems across all chronic health conditions. Analogously, we assume that for brain disorders, despite the overlap with other chronic health conditions, there may well be specific PSD that are more closely linked to these disorders than to other health conditions.

Now that we have evidence in support of the hypothesis of horizontal epidemiology, what are the consequences? We see consequences in clinical practice, research and policy making.

While highly specialized brain-disorder knowledge and specific diagnostic criteria are required for diagnosis, a broader, more cross-cutting approach is necessary to understand the impact of brain disorders in daily life and to develop and implement interventions and strategies that directly address this impact. Applying interventions across brain disorders opens the door to a more efficient health care provision in which health professionals can rely on common clinical experience and knowledge transfer to address similar PSD. The Canadian experience in increasing access to cognitive-behavioural therapy irrespective of the disorder, is an example of how to achieve cost-effectiveness with a horizontal approach [40].

There is also some evidence that a cross-cutting strategy to health care in brain disorders is cost-effective especially in low resource settings where every effort needs to be made to spread limited care resources [41, 42]. The results of the PARADISE project suggest that in the care of persons with brain disorders high resource countries might well learn from the experience of implementing cross-cutting intervention strategies in low resource settings.

In research, we see in light of our results the need for studies that capture the impact of brain disorders horizontally. As noted, Wittchen et al. found no European study that systematically examined impact across brain disorders [3] and in PARADISE we widened that search to include international studies and found the same results (the database for this work can be obtained from the corresponding author). As we have seen with the surprising results of PARADISE, cross-cutting horizontal research in PSD in brain disorders pays off in terms of broadening our understanding of the impact of these disorders in daily life, and on what are important determinants of this impact.

We also see the need for an outcome measure to describe the impact of living with a brain disorder that is sensitive to change over time and able to capture similarities and differences between disorders. This is required to evaluate effectiveness and cost-effectiveness of interventions across brain disorders, as has been pointed out by other authors. The widely-used Short Form-36 [43] and the EQ-5D [44] have been shown not to be sensitive to people with mental disorders [45, 46]. While there are many condition-specific PROs addressing problems in functioning and health-related quality of life in persons with specific brain disorders, there is no generic instrument addressing difficulties that persons with brain disorders experience in their life. The results of the PARADISE project form the ideal basis to develop a generic, brain disorder outcome measure, as recently reported [47, 48].

Health policy-makers, unlike clinicians and researchers, now recognize the importance of brain disorders as a whole, in order to understand the genetic, biochemical and structural impairments behind brain disorders [49]. In addition, a better and broader understanding of PSDs that people with these disorders confront daily is needed. Knowledge about the environmental determinants of those difficulties, as well as the determinants of their deterioration or improvement, can lead to a wide range of interventions that can truly make a difference to people’s lives. Investments in research focusing on the development of social and psychological interventions are urgently needed. People with brain disorders themselves need to hear that exhaustion of medical interventions does not mean that nothing else can be done. The results of this study show that much more can be done to improve the lives of persons with brain disorders with interventions targeting PSDs as well as the environment. As has been shown in previous research, interventions targeting both affected persons and the environment can be extremely effective [50]. Theoretically the scale of the benefit of these interventions can be enlarged if they are implemented across disorders.

This project has limitations that must be considered while interpreting our results. Firstly, it has been carried out from a solely European perspective and data was collected in four European countries, even though the literature reviewed in the preparation phase was international. This may speak against the generalizability of its results that should be validated with data of other regions of the world. Secondly, in some steps of our multi-method approach we relied solely on our research group, for instance to select PROs and salient PSDs. Although all partners participating in the study are acknowledged experts in their areas, we recognize a risk of having prioritized their preferences, and considering a broader expert pool could have led to a different selection of PROs and salient PSDs. We have been, however, very conservative throughout the study and paid attention to include even PSDs and determinants relevant to small group of disorders. Our selection of PSDs and determinants was also critically evaluated by a panel of six external experts, who did not belong to our consortium. Thirdly, it has to be taken into account that the sample included in our study was a convenience one and that the relationship between the severity of a disorder and endorsement rates of PSD and determinants could not be explored, among others. Studies with larger and representative samples, balanced regarding disease severity, are therefore required to confirm our results, and to explore their robustness when disease severity is considered. Fourth, determinants not fulfilling our criteria were access to alcohol and illegal drugs. We acknowledge that these factors involve very sensitive issues that are difficult to address in an interview and that we have here a risk of social desirability bias. Last but not least, and as mentioned before, our criteria for deciding whether the hypothesis of horizontal epidemiology, though intuitive, were nevertheless neither supported by evidence nor verified with any statistical tests. We welcome arguments from readers suggesting more appropriate criteria.

## Conclusion

In conclusion, our study provides first evidence that supports the hypothesis of horizontal epidemiology in brain disorders: Psychosocial difficulties and environmental determinants of those difficulties are experienced in common across brain disorders. This result challenges the brain disorder-specific or vertical approach in which clinical and epidemiological research about psychosocial difficulties experienced in daily life is commonly carried in neurology and psychiatry and the way in which the corresponding health care delivery is practiced in many countries of the world.

## Supporting Information

S1 File Summary PRISMA-like flow chart of the nine systematic reviews. (TIF)

S2 File PRISMA-like flow chart covering the nine systematic reviews. (TIFF)
