# Supplementary figures and images for: Understanding the Impact of Brain Disorders: Towards a ‘Horizontal Epidemiology’ of Psychosocial Difficulties and Their Determinants
Source: PLoS One. 2015 Sep 9;10(9):e0136271. doi: 10.1371/journal.pone.0136271 (PMC4564202; doi:10.1371/journal.pone.0136271)

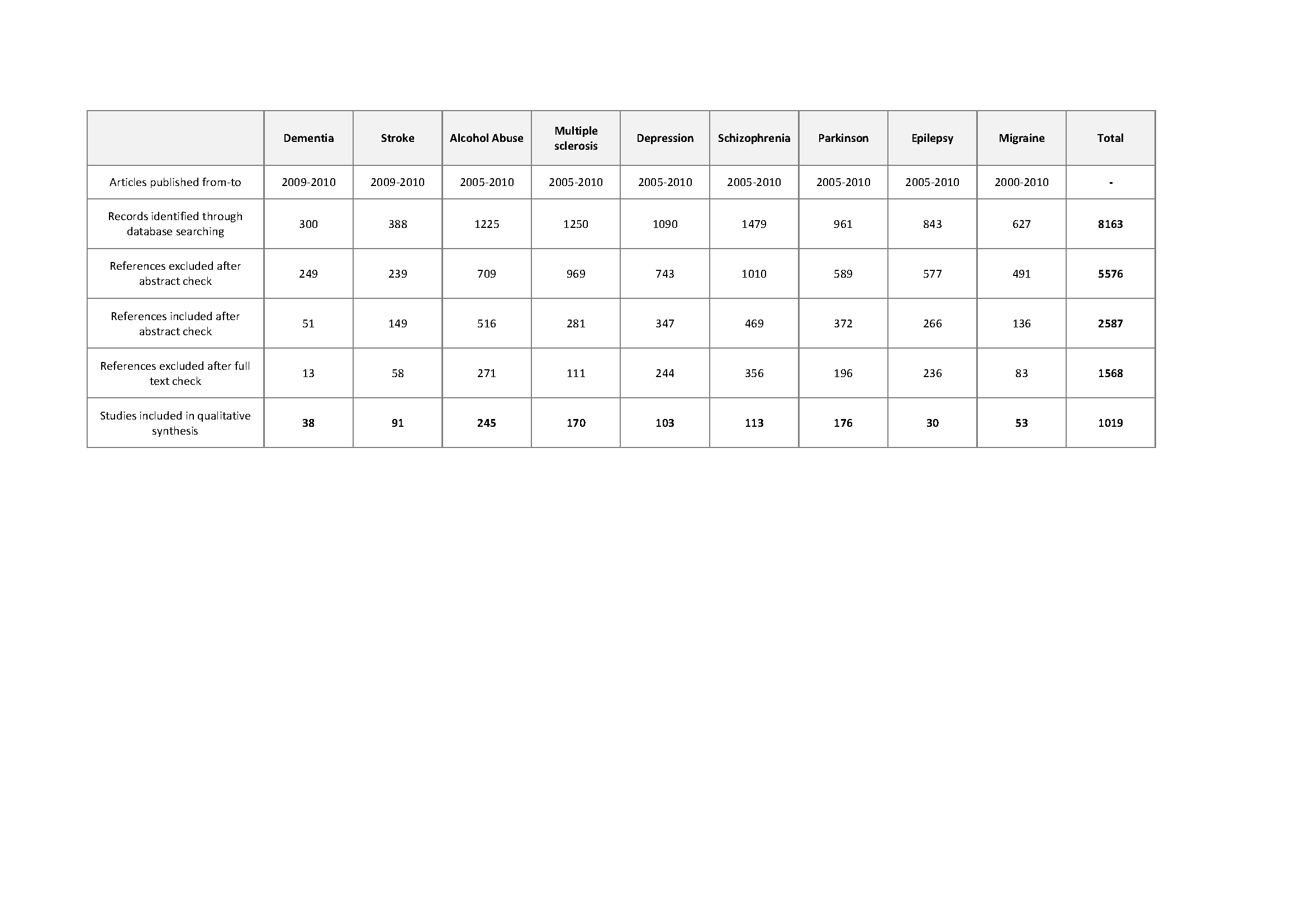

Supplement: S2 File — (TIFF) [file pone.0136271.s002.tiff]
